# Editorial: Physical activity behavior, obesity, and stress as crucial sources of health issues in stressful occupations, volume II

**DOI:** 10.3389/fendo.2025.1742390

**Published:** 2025-11-25

**Authors:** Filip Kukić, Katie M. Heinrich

**Affiliations:** 1 University of Banja Luka, Faculty of Physical Education and Sports, Banja Luka, Bosnia and Herzegovina; 2 Department of Research and Evaluation, The Phoenix, Denver, CO, United States

**Keywords:** occupational health, occupational risks, stress response, first responders, physical inactivity, shiftwork

Occupations at the front line of public safety, security and health (e.g., military personnel, police officers, firefighters, medical professionals, social workers, airline pilots) experience inherently high physical and psychological demands. These demands include a combination of chronic stressors, variable schedules, trauma exposures, physical loads, and interrupted recovery that impact the endocrine and metabolic systems in ways that may predispose individuals to adverse health outcomes such as obesity, metabolic dysfunction, musculoskeletal injury, diminished performance and impaired quality of life. The present Research Topic set out to explore how physical activity behaviours, body composition (especially adiposity and muscle mass), and occupational stress intersect to influence endocrine, metabolic and health outcomes in these stressful professions (1).

## Key themes and highlights from the research topic

Physical Activity Behaviour, Obesity, and Stress as Crucial Sources of Health Issues in Stressful Occupations (Volume II), extends previous work in this field by curating a set of interdisciplinary studies exploring how lifestyle behaviours, body composition, and psychosocial stress converge in high-pressure occupational settings (2–4). The articles span diverse professional groups and research methodologies, yet together they reveal consistent themes that deepen our understanding of the interconnection between stress, physical activity, and obesity.

One group of studies focus on physical activity patterns and determinants among professionals working under demanding conditions. Research in police officers and healthcare personnel emphasize how irregular work hours, long shifts, and psychosocial strain often reduce opportunities and motivation for regular exercise (Feng et al., Xu et al.). These findings highlight how structural aspects of the workplace—rather than individual attitudes alone—shape movement behaviours. Encouragingly, the studies also show that individuals who maintained consistent exercise routines demonstrated greater psychological well-being and reduced indicators of burnout, underlining the protective role of physical activity against occupational stress (Xu et al.).

Other papers address the interplay between occupational stress, body composition, and metabolic risk (Feng et al., de Lira et al.). Longitudinal observations in hospital workers reveal that sustained stress exposure is associated with unfavourable shifts in fat distribution and muscle mass, even when total body weight remains stable (de Lira et al.). Similar trends exist among healthcare professionals in other contexts, where high rates of overweight and obesity correlate with elevated cardiovascular risk factors (Nasution et al.). These findings suggest that metabolic health within stressful occupations is not adequately captured by body weight alone but requires considerations of hormonal and compositional adaptations associated with chronic stress and disrupted energy balance.

Complementary to this, other studies examine psychological mediators and coping mechanisms that link stress, emotion regulation, and physical activity behaviour (Xu et al., Wojczyk et al.). Analyses of urban workers demonstrates that emotional self-efficacy and psychological detachment during off-duty periods play critical roles in buffering against burnout (Xu et al.). Workers who perceived themselves as capable of managing stress were more likely to remain active and report better mental health. These results reinforce the notion that the relationship between stress and activity is bidirectional, such that psychological resilience supports movement behaviour, and regular movement, in turn, enhances resilience through endocrine and neurochemical pathways.

Finally, population-based data explores the associations between adiposity, metabolic dysfunction, and mental health, providing mechanistic context relevant to occupational settings (Liu et al.). Links between visceral fat and depressive symptoms suggest that endocrine pathways, including dysregulation of the hypothalamic–pituitary–adrenal axis and inflammatory signalling, may contribute to both psychological distress and metabolic risk. Such insights emphasize the need to view obesity in stressful professions not merely as a behavioural or nutritional issue but as an endocrine and stress-driven process.

Together, these studies emphasise several converging themes:

1. Even with clear evidence of risk, individuals in highly stressful occupations often maintain low levels of physical activity, limited muscle strength or muscle‐mass preserving activity, and high sedentary behaviours.
2. Adiposity and body composition are modifiable but often under‐addressed factors in occupational health, particularly regarding how visceral fat and muscle mass interact to influence metabolic risk under conditions of stress.
3. Stress operates as both cause and a consequence: chronic occupational stress triggers neuro‐endocrine dysregulation (e.g., HPA-axis, cortisol changes, insulin resistance) and is further exacerbated by poor body composition, physical inactivity and reduced physical capacity.
4. Mechanistic pathways and psychosocial mediators, including emotional self‐efficacy, psychological detachment, burnout, and exploring how physical activity moderates these processes represents a promising direction for future interventions.
5. It is important to consider organisational and environmental context since many of the barriers to healthy behaviours among frontline workers are structural (shift work, workload, recovery time, lack of facilities, cultural norms), such that individual behaviour change alone may be insufficient.

Across these contributions, several consistent messages emerge. First, chronic stress is both a cause and a consequence of poor physical health in demanding professions. Second, physical activity functions as a critical regulator of endocrine balance, emotional stability, and metabolic control, yet its promotion requires organizational commitment, not just individual motivation. Third, body composition, and visceral adiposity in particular, represents a sensitive indicator of the cumulative physiological toll of stress and inactivity. Finally, psychological and environmental supports are essential to break the self-perpetuating cycle of stress, inactivity, and obesity.

Looking forward, future investigations should aim to clarify the biological mechanisms linking occupational stress to metabolic outcomes, quantify dose–response relationships between activity and stress biomarkers, and evaluate interventions that align with the unique temporal and psychological realities of high-stress professions. Longitudinal, interdisciplinary approaches that combine endocrinology, physiology, psychology, and occupational health are crucial for advancing both theory and applied practice.

## Implications for practice, policy and future research

From a practice standpoint, this Research Topic reinforces that interventions in stressful occupational cohorts should not merely adapt general‐population physical activity recommendations but rather account for the unique demands, schedules and recovery limitations of these professions. For example, strength training and maintenance of muscle mass may carry particular importance given the physical tasks often required in tactical or emergency professions.

From a policy and organisational perspective, the findings suggest that workplace health promotion must integrate: (a) scheduling and recovery optimisation; (b) access to strength/aerobic training opportunities; (c) support for stress mitigation (including psychological detachment, emotional regulation, resilience); and (d) body‐composition monitoring and intervention (beyond simple body mass index). Holding organisational leadership accountable for workforce‐health metrics may be an important lever.

This Research Topic opens several promising avenues for future research. These include deeper mechanistic studies of endocrine, immune, and metabolic pathways linking stress, physical activity and adiposity; longitudinal tracking of occupational cohorts over extended periods; intervention trials tailored to specific high‐stress occupations; and multilevel studies integrating individual, interpersonal and organisational factors. The endocrine systems (e.g., HPA axis, adrenals, muscle‐myokine signalling) offers a central focus for such integrative work.

## Conclusion

In stressful occupations, the triad of inadequate physical activity, unfavourable body composition (especially obesity/adiposity) and persistent stress creates a powerful and interlocking threat to health, performance and quality of life. The papers gathered in this Volume II of our Research Topic advance our understanding of how these elements interrelate, particularly from an endocrine and metabolic lens, and point towards actionable strategies for change. It is our hope that this Research Topic will encourage the translation of evidence into practice, inspire organisational change, and catalyse further research that empowers frontline workers to thrive physically, mentally and professionally.
